# The Book World of Medicine and Science

**Published:** 1896-03-28

**Authors:** 


### THE BOOK WORLD OF MEDICINE AND SCIENCE.

The Medical Annual, 1896. (Bristol: John Wright and Co. Price 7s. 6d. net.)

"The Medical Annual" this year again maintains its reputation as a convenient book of reference for the medical practitioner. It differs from the "Year-book of Treatment," in that, in addition to a general sketch of new remedies, &c., it contains original articles on various matters of interest. Some of these are very good, and greatly relieve what might otherwise be the monotony of a mere volume of condensed reports. Among them we may mention a very full article on "Angio-Neuroses," by Ramsay Smith; one on "The Remedial Value of Cycling," by Oscar Jennings; some most useful coloured plates showing "The Sensory Distribution of Spinal Nerve Roots," by William Thorburn; an article on "Life Assurance," by De Havilland Hall; and various articles on "Diseases of the Nose and Throat," including a good summary of what is known about "The Antitoxin Treatment of Diphtheria," by Watson Williams. Many other articles might be mentioned, among which we may more especially refer to the various contributions on "Abdominal Surgery," by Mayo Robson, and to the very careful survey of "General Surgery," given by Priestley Leech in many articles scattered throughout the book. It is, on the whole, a very satisfactory volume.

